# Supplementary material for: Chromothripsis during telomere crisis is independent of NHEJ, and consistent with a replicative origin
Source: Genome Res. 2019 May;29(5):737–49. doi: 10.1101/gr.240705.118 (PMC6499312; doi:10.1101/gr.240705.118)
Supplement: Supplemental Material [file supp_gr.240705.118_Supplemental_file_1.zip › contigs/annotated_contigs/DB92/contig.2.DB92_length_733_mean_cov_6.95770804911.docx]

**DB92_length_733_mean_cov_6.95770804911**

```
CCTGAACCCAGGAGGTGGAGGTTGCAGTGAGCCGAGATCACGCCACTGCACTCCAGCCTGCCAACAGAGTGAGAATCCGTCTGAAAAAA
 >chr11:9892436-9892769 + E=7e-179
AAAAAAGTAACTCAAGATGGATTACAGACTAAAATGTAAAATGCAAAACTATCAAAAACCCTAGGAGAAAATCTAGGCAATACTTTTCA

GGATATAGGCATGAGCAAAGATTTCATAATGAAAGCACCAAAAGCAATTGCAACAAAAGCAAAACTGACAAATGGGATCTAATTAAACT

AAAGAGCTTCTGCACAGCAAAAGAAACTATCATCAGAGTGAACAGAAAACCTACAGAATGG|TTTTACA|CTGTTAATCACTTTGGGAG
                                                                     >chr11:9758499-975
GACAGGGTGGGAGGATCACTTGAGGCCAGGAATTTGAGACCAGCCTGGGCAACATAGCGAGACCTCATAGCAGTCAACCAACAAACCAA
8897 + E=1e-227
CAACACTTTTAGCTGGTCCGTGTGTTAGTGTGTTGTTCTCATGGAGCTTCTGGCCTCCTCTTAATTCTCAGCCACTCTTGCCTGGAATA

AAGTTTCTGCAACATGCAGCTGGGGACAAAGAGAAATTCTGGCAGCCAGCCTGCCCCTCCCAGGAAGGTATCATAGCCCTAGGCTTGAG

CTGAGGGGAGGGGAACCCTTGTTTGGGGTTCCATCTGCCAGGAGAAGAGCTTTCTTCTCACTGACACTGTGGGGGTGGGGAGGGAGTGC

ATCATATGCCACAGACCCTCACG
```
